# Rhizobacteria Communities of Phytoremediation Plant Species in Petroleum Hydrocarbon Contaminated Soil of the Sudd Ecosystem, South Sudan

**DOI:** 10.1155/2020/6639118

**Published:** 2020-12-24

**Authors:** J. A. Ruley, J. B. Tumuhairwe, A. Amoding, O. T. Westengen, H. Vinje

**Affiliations:** ^1^Department of Agricultural Production, Makerere University, P.O. Box 7062, Kampala, Uganda; ^2^Department of Agricultural Sciences,CNRES, University of Juba, P.O. Box 82, Juba, Sudan; ^3^Department of International Environment and Development Studies (Noragric), Norwegian University of Life Sciences (NMBU), Ås, Norway; ^4^Department of Chemistry, Biotechnology and Food Science, Norwegian University of Life Sciences (NMBU), Ås, Norway

## Abstract

The Sudd wetland is one of the oil-rich regions of South Sudan where environmental pollution resulting from oil extraction activities has been unprecedented. Although phytoremediation is the most feasible technique, its efficacy reduces at high TPH concentration in soil. This has made rhizoremediation the most preferred approach. Rhizoremediation involves use of a combination of phytoremediation and biostimulation. The process is catalyzed by the action of rhizobacteria. Therefore, the objective of this study is to characterize rhizobacteria communities prevalent in phytoremediation species growing in hydrocarbon-contaminated soils biostimulated with cattle manure. The treatments studied were plant species only (T1), plant species and hydrocarbons (T2), plant species and manure (T3), and plant species, manure, and hydrocarbons (T4). The rhizobacteria communities were determined using pyrosequencing of 16S rRNA. In the treatment with phytoremediation species, hydrocarbons 75 g · kg^−1^soil, and cattle manure 5 g · kg^−1^soil (T4), there was a significant increase (*p* < 0.05) in rhizobacteria abundance with the highest ASV observed in *H. rufa* (4980) and the lowest in *S. arundinaceum* (3955). In the same treatment, bacteria community diversity was high in *H. rufa* (Chao1, 10310) and the least in *S. arundinaceum* (Chao 1, 8260) with Proteobacteria, Firmicutes, and Actinobacteria as the dominant phyla. Similarly, in contaminated soil treated with cattle manure, there was a significant increase (*p* < 0.05) in abundance of rhizobacteria genera with *Pseudomonas* dominating across phytoremediation species. *H. rufa* was dominated by *Bacillus, Fusibacter,* and *Rhodococcus*; *G. barbadense* was mainly associated with *Luteimonas* and *Mycobacterium,* and *T. diversifolia* was inhabited by *Bacillus* and *Luteimonas*. The rhizosphere of *O. longistaminata* was dominated by *Bacillus, Fusibacter,* and *Luteimonas,* while *S. arundinaceum* was largely inhabited by *Sphingomonas.* These rhizobacteria genera ought to be applied in the Sudd region for bioremediation.

## 1. Introduction

Globally, crude oil is a critical resource for national development. The world greatly depends on oil and, as a result, vast amount is used, transported, and stored [1]. Crude oil is number one source of energy and a primary raw material for major industries worldwide [2].Oil remains an indispensable input for sectors such as manufacturing (as a raw material and fuel), transport (fuel), and trade (as an export commodity), and there has been a steady increase in global demand for crude oil over the last decades [2, 3, 4]. Over the next two decades, Rada and colleagues [5] anticipate that world oil demand could even rise to 170 million barrels per day. This belief has compelled producing countries to devise ways and means of increasing production, transportation, and refining of crude oil in order to meet the purported growth in demand [2, 4], South Sudan inclusive [6].

South Sudan is one of the famous crude oil producing countries falling 3^rd^ after Nigeria and Angola in Africa [6] and 83^rd^ among the 171 oil producing countries worldwide [7]. Oil is the lifeline of its economy for now and over the medium term [6]. For example, between 2008 and 2011, oil exports accounted for 98% of government revenue [6]. This position has fundamentally changed her economy from agriculture to industrialization. South Sudan has a production capacity of 298,000/390,000 barrels per day [8, 9] including Sudd wetland. Sudd is the largest wetland in the whole world and covers 57,000 Km^2^ that makes up approximately 5% of the total land area of the Republic of South Sudan (648,000 Km^2^) [10, 11]. The area of the wetland is larger than countries such as Switzerland, Belgium, the Netherlands, and Singapore [12]. Owing to this enormous size, the Sudd ecosystems are of vast socioeconomic, cultural, and biological importance locally, nationally, and internationally. This accounts for why it was designated as a Ramsar site in 2006 [10] making it an essential nature conservation area.

In all crude oil producing countries around the world, during the process of crude oil production and secondary activities such as transportation and storage, several solids, liquids, and gaseous forms of wastes and pollutants are generated [13]. Also, spills and discharges of petroleum hydrocarbons (PHC) in some environments have reportedly been caused by initial activities such as oil field development, transportation activities such as leakages from oil pipelines and haulage tankers, oil well waxing, and at times when refining and petrochemical equipment is being overhauled [14–16] contributing to gross contamination of ecosystems.

Soil contamination with PHC is a widespread problem and has hazardous implications on both environment and human health [17, 18]. As earlier stated, Sudd wetland is an oil rich zone. As a result, the Sudd ecosystems are fragile and therefore threatened by oil exploration and extraction activities since the 1980s [19–21] with notable effects on the environment and natives of the area. The commonly reported effects by studies [20–22] include high salt content in water, death of livestock, reduction in vegetation cover, and outbreak of strange diseases hitherto uncommon among people in the local communities.

Attempts to remediate hydrocarbon-contaminated soil are a priority in national development plans of many oil-producing countries in order to counteract the harmful effects of PHC [23, 24]. South Sudan is not exceptional. The techniques deployed in remediation of PHC polluted soil are fivefold: chemical, physical, electrical, thermal, and biological [25, 26]. The use of chemical treatment involves chemical precipitation, membrane separation, ion exchange, carbon absorption, aqueous chemical oxidation, and surfactant enhanced recovery [25]. With regard to physical treatment, the main treatment measures involve land filling, pumping and treating, dual phase extraction as well as air sparging, and dual phase extraction [25, 26]. For electrical remediation, electrical principles are applied to decontaminate particular sites though only limited to granular type of soil contaminated with heavy metals [25]. The thermal methods are largely used in environments where the contaminants are highly volatile and include *in situ* vitrification, incineration, and electrical pyrolysis. However, the above-mentioned techniques are not largely used at present because they are associated with various shortcomings such as high cost, handling of the generated excess waste, and secondary contamination [25, 26]. This has given way to biological remediation techniques. It is not by surprise therefore that in recent times, in most crude oil producing countries, biological remediation techniques are dominating any efforts for ecorestoration of PHC contaminated soils [27], bioremediation inclusive [25].

Bioremediation-based rhizoremediation is a biological technology with great potential of restoring PHC contaminated soils [28, 29]. Rhizoremediation refers to a process in which the PHC contaminants are degraded by bacteria in the rhizosphere [23, 24] and uses a suite of indigenous microorganisms [30]. This approach is nondestructive and is environmentally acceptable [28, 29, 31] making it a desirable and sustainable technique [31]. For example, it does not generate toxic metabolites [29]. Relatedly, Shukla and colleagues [32] contend that the mechanism provides a natural corrective solution in which the synergy between roots of phytoremediation species and resident plant growth promoting rhizobacteria (PGPR) boosts secretion of root exudates, production of siderophores, phytohormones, and phosphatases. This accounts for why the approach is regarded an effective natural remedy for ecorestoration of polluted sites [32] leading to its popularity as a green technology as reported elsewhere [33–35].

Compared to other soil inhabiting microorganisms, bacteria are the most dominant and, therefore, primary microbial communities that play a fundamental role in biodegradation of PHC contaminants. The various genera utilize hydrocarbons as carbon and energy sources [36]. The degradation potential of bacteria is harnessed with biostimulation using manure. For example, cattle manure improves soil physicochemical characteristics, hence enabling adaptability of bacteria in contaminated soil [37]. Additionally, some manure compounds (such as nitrogen, phosphorus, and potassium) are high-energy electron acceptors and sources of nutrients for bacteria. Although the degradation potential of different bacteria varies [38], studies [39, 40] have reported *Acinetobacter, Pseudomonas, Gordonia, Rhodococcus, Immundisolibacter, Luteimonas, Alcanivorax, Marinobacter, Mycobacterium, Corynebacterium, Bacillus, Ochrobactrum, Sphingomonas,* and *Hahella* as the most active hydrocarbon degrading genera in rhizosphere. However, the abundance of these genera in the rhizosphere of contaminated soils in the Sudd ecosystems is unknown yet; it is essential for harnessing bioremediation. Furthermore, Mackova and colleagues [41] have shown that inoculation has limited efficiency to biodegrade hydrocarbons due to incompatibility between bacteria and plant species. The deployment of efficient PHC biodegrading bacteria in contaminated soils needs to be coupled with the use of phytoremediation species that enhance their survival and growth [42, 43]. Thus, detailed characterization of rhizosphere bacteria is necessary to facilitate selection and use of efficient PHC biodegrading species of bacteria for inoculation during phytoremediation [44]. Therefore, the objective of this study was to determine the rhizobacteria communities influenced by phytoremediation species growing in cattle manure biostimulated crude oil contamination soils in the Sudd region using sequences of 16S ribosomal RNA.

## 2. Materials and Methods

### 2.1. Experimental Design

A screen house pot experiment was conducted at Makerere University Agricultural Research Institute, Kabanyolo (MUARIK), from January 2018 to April 2018 (120 days). The soil used in the experiment was collected from nonhydrocarbon contaminated natural undisturbed land in the Sudd region as composite topsoil samples at a depth of 0–30 cm. Treatments included two rates of partially decomposed (1.7 : 0.6 : 0.8 NPK) cattle manure, two rates of crude oil, and five phytoremediation plant species arranged in Completely Randomised Design (CRD). Cattle manure was applied at rates of 0 and 5 g·kg^−1^ soil confirmed as economically appropriate [45], while crude oil rates 0 and 75 g · kg^−1^ soil were used. The phytoremediation plant species were (i) wild cotton (*Gossypium barbadense*), (ii) Sudan grass (*Sorghum arundinaceum*), (iii) wild rice (*Oryza longistaminata*), (iv) false sunflower (*Tithonia diversifolia*), and (v) thatching grass (*Hyparrhenia rufa*). These plant species are abundant in the Sudd region [46] and were screened and confirmed as suitable for phytoremediation [47].

In this study, polypropylene plastic-made pots were used. The soil was apportioned into 5 kg pots. To ensure that soil, cattle manure, and crude oil are mixed thoroughly, a metallic sheet was used as a mixing base. The mixture was returned into the pots with perforated bases to allow aeration. Each pot was labeled with the name of the respective treatment. The labeled pots were left for one week before planting. To cater for any PHC losses, pots were placed on their lids. Any water leachate was used to irrigate the respective pot. The lids were also washed after every two days and the wash water used to irrigate respective pots.

At 120 days after planting, roots were removed from the pots. The firmly attached soil to the roots was collected from all pots (60 treatments) and transported in an ice cooler to the Biotechnology Laboratory of Makerere University Regional Centre for Crop Improvement at Kabanyolo, Wakiso district, Uganda, and stored at −80^o^C until extraction of genomic DNA.

### 2.2. Molecular Analysis of Bacterial Communities

Genomic DNA was extracted from 0.25 g of the rhizosphere soil sample using DNeasy Power Soil® DNA Isolation Kit (Qiagen Company) following the manufacturer's instructions. Bacterial diversity was analysed using culture independent molecular technique16S rRNA gene PCR. The primers used in PCR reactions were 341F and 785R. A GC clamp was added to forward primer (F). These targeted approximately 300 bp of hypervariable V3 region. All PCR amplifications were performed using ThermoHybaid PCR cycler (Molecular Biology Instrumentation, Massachusetts, USA). PCR mixtures were prepared with 5 *μ*l of Taq buffer 10×, 2.5 mM of MgCl_2_, 200 *μ*mol of each deoxynucleoside triphosphate (dNTP), 20 pmol each primer, 5 *μ*g of bovine serum albumin, 1% of formamide and 2.5U Taq polymerase (Roche Molecular Biochemical, Mannheim, Germany), and sterile filtered Milli-Q water to a final volume of 50 *μ*l. The PCR program was as follows: denaturing step of 94°C for 3 min, followed by 35 cycles of 1 min at 94°C, annealing for 1 min at 55°C, and elongation for 1 min at 72°C, followed by a final elongation at 72°C for 10 min.

The concentrations used for PCR were as follows: total mixture, 25 *µ*l; dNTPs, 50 *µ*M; genomic DNA, 30 ng/*µ*l; and each primer, 10 pmol/*µ*l. The concentration of MgCl_2_ in the reaction mixture was maintained at 1.5 mM for effective amplification. MgCl_2_ was a cofactor for Taq enzyme and helped in adding correct dNTPs complementary to the sequence in newly synthesizing strand by binding to dNTPs. A second PCR reaction was performed using 5 ml of the first PCR products as template under the same primers (GC clamp attached to the primer U968f) and conditions specified for the first PCR reaction. The PCR reactions were performed in duplicate, in order to obtain adequate DNA amount for electrophoresis. Amplification products were checked in 1.3% agarose gels stained with ethidium bromide (0.1 mg/ml). This was followed by storage at −20°C and then sending to the LGC Genomics Sequencing Centre in Germany. Purified PCR products were pyrosequenced using Illumina MiSeq by the LGC Genomics Sequencing Centre in Germany (http://www.support.illumina.com/downloads/bcl2fastqconversion-software-v2-19.html).

### 2.3. Data Preprocessing

After sequencing, demultiplexing of all libraries for each sequencing lane was done using Illumina bcl2fastq 2.17.1.14 software (folder RAW). One to two mismatches were allowed in the barcode and read when the barcode distances between all libraries on the lane allowed for it. The sorting of reads by amplicon inline barcodes (folder RAW) was done through one mismatch that was allowed per barcode. The barcode sequence was then clipped from the sequence after sorting and reads with missing barcodes, one-sided barcodes, or conflicting barcode pairs were discarded.

Clipping of sequencing adapter remnants from all reads (folder AdapterClipped) was carried out and reads with final length < 100 bases were discarded. The primer detection and clipping (folder Primer Clipped) was done by allowing three mismatches per primer; pairs of primers (Fw-Rev or Rev-Fw) were present in the sequence fragments. Whenever primer-dimers were detected, the outer primer copies were clipped from the sequence. The sequence fragments were turned into forward-reverse primer orientation after removing primer sequences.

### 2.4. Bioinformatics Processing

After primer removal and clipping of sequences, read sequences were loaded into R (version 3.6) and run through DADA2 pipeline (version 1.12) [48]. The sequences were filtered and trimmed using “*filterAndTrim*” function. The trimming specifications were as follows. First, truncation length (truncLen) was set to 250 bases for both forward and reverse reads. Secondly, cutoff for maximum expected error calculated from the quality score (maxEE) was set to 3 for both forward and reverse reads for quality plots. The remaining parameters were held as default. The error rate was estimated by function “*learnError*.” Thirdly, a dereplication process was conducted with function “*derepFastq*.” All identical sequencing reads were combined into one unique sequence with a corresponding abundance equal to number of reads with that unique sequence.

Before merging, core sample inference algorithm was applied to data [49]. The forward and reverse reads were then merged together to obtain full denoised sequences with function “*mergePairs*.” As defaults in DADA2, merged sequences were only output if forward and reverse reads overlapped with a minimum of 12 bases. The merged sequences were then rearranged in an Amplicon Sequence Variant (ASV) table [50] and cleaned for chimeras with functions “*makeSequenceTable*” and “*removeBimeraDenovo,*” respectively. For taxonomic classification, recommendations of Callahan and colleagues were used [50], together with a native implementation of Naïve Bayesian classifier method [51] using function “*assignTaxonomy*” still in DADA2 package. The ASVs with chimeras were removed from analysis using “*subset*_*taxa*” function in “*Phyloseq*” package (https://github.com/joey711/phyloseq).

### 2.5. Statistical Analysis

All statistical analyses were performed in R software (V2.15.3). To estimate coverage and sampling diversity, rarefaction curves were constructed. “*Phyloseq*” package calculated population diversity (Simpson index), evenness (Shannon index), and richness (Chao1). To test effect of treatments on bacterial community structures, PERMANOVA analysis using “*adonis*” function in “*vegan*” package was performed. To ensure that PERMANOVA results were not affected by in-group dispersions, an analysis of multivariate homogeneity of group dispersion was conducted for different treatments using “*betadisper*” function in “*vegan*” package (Anderson, 2006). Differences in bacterial community dispersion between treatments were assessed using PERMDISP, since a significant PERMANOVA result may indicate either a difference in centroids or an unequal dispersion between treatments. Multivariate analysis using nonmetric multidimensional scaling (nMDS) and principal component analysis (PCA) were used to explore hierarchical structure of bacterial community composition under effects of different treatments. These were calculated from Bray–Curtis matrices using the “*metaMDS*” function from “*vegan*” package.

## 3. Results

### 3.1. Effect of Treatment on Bacterial Community Richness

**Figure 1:**
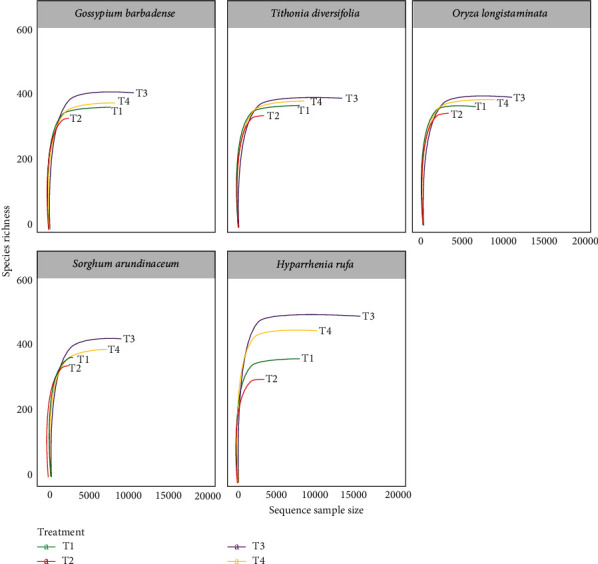
Rarefaction curve showing sampling depths across the five plant species with and without compost and petroleum contamination (T1 = plant species only, T2 = plant species and hydrocarbon, T3 = plant species and Manure, and T4 = plant species, manure, and hydrocarbon).

A total of 5 million high-quality paired-end reads were generated from Illumina MiSeq platform with an average of 83,333 reads per sample (*n* = 60). The tags were obtained with a maximum of 81,480 filtered sequences clustered to 3927 amplicon sequence variants (ASVs) of sixty samples at 3% confidence interval. Rarefaction was conducted to approximate the number of ASVs in random samples. The rarefaction curves (Figure 1) asymptotically approached a plateau, suggesting that the curves accurately reflected microbial community richness and indicated that the sequencing efforts were sufficient for this study.

PERMANOVA analysis showed cattle manure and hydrocarbon contamination significantly affected rhizobacteria community but not plant species (Table 1). The interaction of plant species and cattle manure explained 5.1% of ASVs variation in community structure. Similarly, interaction of plant species and hydrocarbon accounted for 9.2% of variation in ASVs. Biostimulation of plant species for bioremediation of TPH contaminated soil with cattle manure explained 13.4 % variation in assemblages of bacterial communities (Table 1).

### 3.2. Taxonomic Bacterial Community Composition

The sequences were classified into 33 phyla, 54 classes, 128 orders, 268 families, and 511 genera of bacteria at 80% bootstrap. Treatments containing plant species and manure (T3) had the highest number of phyla and genera. This was followed by treatments containing plant species, manure, and hydrocarbon (T4), while plant species and hydrocarbon (T2) had the least number of phyla and genera. The most and least abundant rhizobacteria communities were noted in treatments with plant species, manure and hydrocarbon (T4) and plant species and hydrocarbon (T2), respectively. Similarly, most and least diversities were observed in T4 (*H. rufa* and *T. diversifolia*) and T2 (*S. arundinaceum*), respectively (Table 2).

### 3.3. Bacterial Community Abundance and Diversity

The richness and diversity of bacterial communities significantly differed (*p* < 0.000) between plant species in all tested parameters except Simpson's index (Table 2). In all the five-plant species, there was high abundance of bacterial communities in the treatment with plant only (T1). However, when TPH was added to plant species (T2), there was a significant (*p* < 0.05) decline in abundance as shown by a drop in Chao 1 values across the five phytoremediation species. Addition of cattle manure (T4) to the treatment (plant + TPH) significantly increased (*p* < 0.05) bacterial abundance. In *S. arundinaceum*, the communities quadrupled (from 2097 to 8260); in *G. barbadense*, there was multiple increase (from 2781 to 9540) and a near multiple increase in *O. longistaminata* (from 3597 to 9168) and *H. rufa* (from 4304 to 10,310), while in *T. diversifolia*, the communities doubled (from 4223 to 9795).

**Figure 2:**
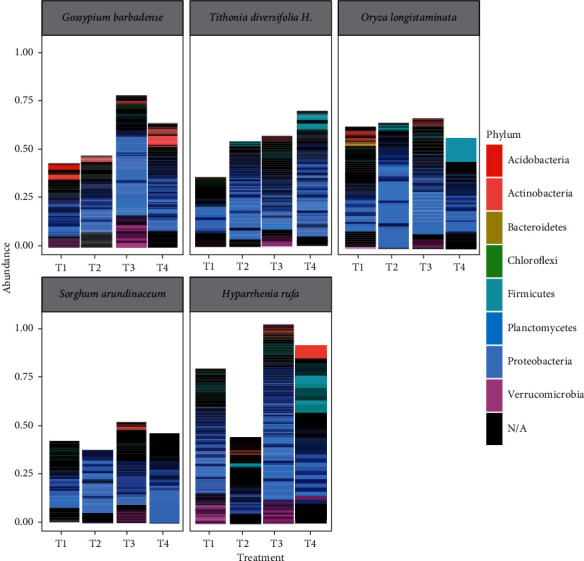
Composition and distribution of bacterial phyla with ≥3% relative abundance across five plant spp. with and without compost and petroleum contamination (T1 = plant species only, T2 = plant species and hydrocarbon, T3 = plant species and manure, and T4 = plant species, manure, and hydrocarbon).

The sudden rise in abundance of bacteria communities in the treatment with plant species, hydrocarbon, and manure (T4) was attributed to addition of cattle manure. Largely, manure improves soil physicochemical properties leading to improved conditions for plant and microbial growth. Therefore, the rhizosphere became a hotspot for survival of different bacteria communities thereby accounting for the increased abundance. Moreover, cattle manure contains bacteria strains, which could have enhanced biodegradation of the TPH. In all phytoremediation plant species, there were significant differences (*p* < 0.05) in diversity in the Shannon index. However, the diversity in the Simpson index was not significant (*p* < 0.05) across all treatments. The most abundant phylum was Proteobacteria across all plant species with or without manure and the TPH accounting for about 41.6% of all ASVs, followed by Actinobacteria (12.7%) and Firmicutes (9.8%), of all ASVs (Figure 2).

Proteobacteria, Firmicutes, and Actinobacteria dominated TPH contaminated soils across all plant species (Figure 2). For example, *G. barbadense* was mainly associated with Proteobacteria and Actinobacteria. The rhizospheres of *T. diversifolia* and *O. longistaminata* were inhabited by Proteobacteria and Firmicutes. Similarly, the rhizosphere of *S. arundinaceum* was dominated by only Proteobacteria, while the roots of *H. rufa* had a high abundance of Proteobacteria, Firmicutes, and Actinobacteria.

### 3.4. Rhizobacteria Genera

**Figure 3:**
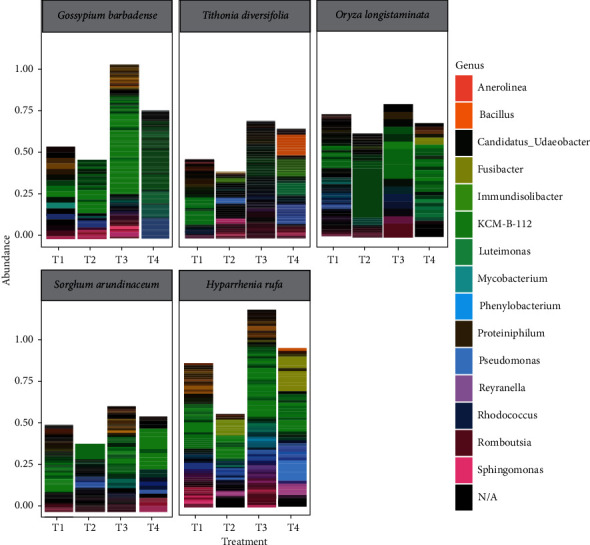
Variation in bacterial genera within rhizosphere of five phytoremediation species under four different treatments (T1 = plant species only, T2 = plant species and hydrocarbon, T3 = plant species and manure, and T4 = plant species, manure, and hydrocarbon).

The dominant genera across all phytoremediation species were *Pseudomonas* (Figure 3). However, it was more dominant in *H. rufa*. Generally, besides *Pseudomonas,* other genera observed in all phytoremediation species were *Luteimonas, Sphingomonas, Mycobacterium, Bacillus,* and *Fusibacter.* These were relatively more abundant in manure treated hydrocarbon-contaminated soil (T4) (Figure 3). Compared to the rest, *H. rufa* had more *Bacillus*, *Fusibacter,* and *Rhodococcus.* Plant species *G. barbadense* was mainly associated with bacteria genera *Luteimonas* and *Mycobacterium,* while *T. diversifolia* was inhabited by *Bacillus* and *Luteimonas*. The rhizosphere of *O. longistaminata* was dominated by *Bacillus, Fusibacter,* and *Luteimonas,* while S*. arundinaceum* was largely inhabited by *Sphingomonas.* There was an increase in bacterial diversity in manure treated hydrocarbon contaminated soil due to addition of cattle manure (Figure 3).

### 3.5. Environmental Influence on Composition of Bacterial Communities

**Figure 4:**
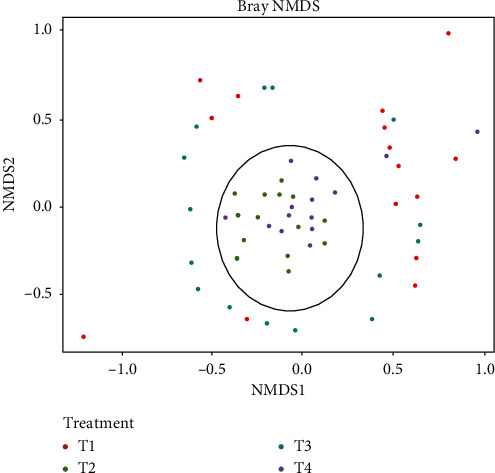
Nonmultidimensional scaling (NMDS) showing ASVs between hydrocarbon (T2, T4) and nonhydrocarbon (T1, T3) treatments. T1 = plant species only (*G. barbadense, H rufa T diversifolia, O longistaminata.* and *S. arundinaceum*), T2 = Plant species and hydrocarbon (0, 75 g·kg^−1^), T3 = Plant species and manure (0, 2 tha^−1^), and T4 = Plant species, manure, hydrocarbon.

Bray–Curtis distance nonmetric multidimensional scaling (NMDS) revealed differences in composition of bacterial communities between hydrocarbon and nonhydrocarbon treatments. In two hydrocarbon-contaminated treatments, one was with plant species and hydrocarbon (T2) and the other with plant species, hydrocarbon, and manure (T4), where bacterial communities clustered in groups (see the ring in Figure 4). This was different from nonhydrocarbon treatments: one with plant species only (T1) and the other with plant species and manure (T3) where bacterial communities were scattered (Figure 4).

**Figure 5:**
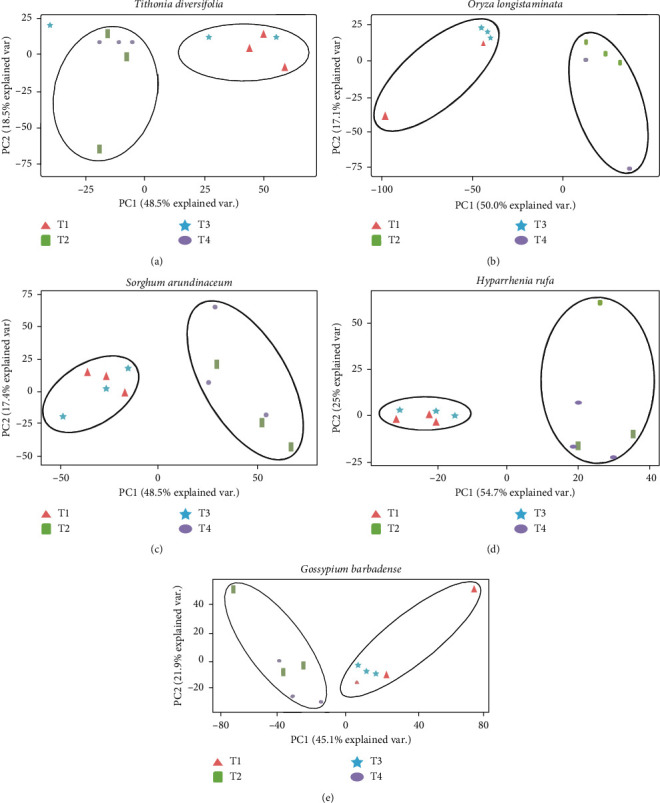
Principal component analysis of bacterial communities across different phytoremediation species growing under different treatments (T1, plant species only; T2, plant species and hydrocarbon; T3, plant species and manure; T4, plant species, manure, and hydrocarbon).

Results from principal component analysis (PCA) also revealed separate clustering of bacterial communities between hydrocarbon and non-hydrocarbon-containing treatments. Irrespective of phytoremediation plant species, bacterial communities in the treatment containing plant species and hydrocarbon (T2) and one containing plant species, hydrocarbon, and manure (T4) clustered separately from one with plant species only (T1) as well as one with plant species and manure (T3) (Figure 5). However, the pattern of clustering was influenced by specific phytoremediation species, a factor explained by differences in percentage variances for PC1 and PC2 (Figure 5).

## 4. Discussion

There were significant variations (*p* < 0.000) between rhizobacterial communities of phytoremediation species with and without hydrocarbon contamination. In the treatments with plant species alone (T1), high bacterial diversity was noted. However, when hydrocarbons were introduced (T2), there was a great reduction in diversity. The bacterial community shifts and eventual decrease in richness resulted from perturbations that normally occur in hydrocarbon contaminated soil. Past studies [52, 53] have proved that introduction of PHC in soil reduces bacterial diversity considerably regardless of the soil matrix type. Nevertheless, in this study, certain bacterial strains resilient to toxicity of PHC existed. These must have used TPH as a source of energy, carbon, or electron receptors for growth. As reported earlier, across all plant species, bacteria genera *Luteimonas, Pseudomonas*, and *Sphingomonas* (phylum Proteobacteria)*, Mycobacteria* and *Rhodococcus* (phylum Actinobacteria) and genera *Bacillus* and *Fusibacter* (phylum Firmicutes) were abundant in the treatment with plant and hydrocarbon (T2) and one with plant species, hydrocarbon, and cattle manure (T4).

Similarly, bacterial communities in hydrocarbon-contaminated soil were significantly affected by biostimulation with cattle manure (T4). The inclusion of cattle manure posted both direct and indirect benefits for the survival of bacterial communities. Directly, cattle manure amendments improved soil physicochemical characteristics enabling speedy adaptation by microorganisms. Furthermore, the introduction of cattle manure must have increased on soil fertility by adding soil organic carbon (SOC), total nitrogen (TN), and NPK. This must have improved plant resilience and performance in the PHC contaminated soil. Accumulation of soil organic carbon for example not only results in increased microbial biomass but also affects microbial community structure and functional diversity [54]. Therefore, cattle manure indirectly influenced a spectacular increase in the microbial diversity observed. Cattle manure additions also improved soil pH and physical properties (aggregation and porosity), thus creating favorable growth conditions for microbes. Earlier studies [55–57] have shown that addition of organic manure to hydrocarbon-contaminated soil enhances multiplication of bacteria population. Furthermore, addition of cattle manure improves soil fertility, which is vital for sustained plant growth [56].

In the rhizosphere, the bacterial colonize the root surfaces, compete against other microbes and form synergestic interactions with host plants [58]. Phyla Proteobacteria, Actinobacteria, and Firmicutes dominated the treatment with hydrocarbon contaminated soil and plant species (T2) and treatment T4 with plant species and manure treated hydrocarbon contaminated soil (T4). These phyla contain members of organotrophic microorganisms that utilize a wide range of organic substrates perhaps including hydrocarbon. Although bacterial strains survive best in aerobic conditions, the three phyla also thrive well in anaerobic environments. Their survival in anaerobic conditions is guaranteed by secretion of intracellular and extracellular enzymes which help in biodegradation of recalcitrant and organopollutants. These bacteria have enzymes capable of assimilating, degrading, and utilizing different hydrocarbon constituents as sources of carbon and energy [29, 58].

Assimilation is a complex biological oxidation process enhanced by supplementation with fixed nitrogen, phosphate, and other nutrients [58]. For example, one of the enzymes, oxidoreductases, enables oxidative coupling to take place enabling both phyla to extract energy via energy-yielding biochemical reactions which cleaves chemical bonds, assisting transfer of electrons from a reduced organic substrate (donor) to another chemical compound (acceptor). In this process, contaminants are finally oxidized to harmless compounds. This guarantees survival of the bacteria communities in a less toxic environment. Furthermore, oxidoreductases catalyze humification of various phenolic substances in soil environment through polymerization and copolymerization with other substrates [59].

The three phyla are also known for secreting oxygenases. In the test samples, oxygenases both monooxygenases and dioxygenases could have been secreted by the phyla. Monooxygenases catalyze desulfurization, dehalogenation, denitrification, ammonification, hydroxylation, biotransformation, and biodegradation of various aromatic and aliphatic compounds, while dioxygenases introduce molecular oxygen into their substrate [60]. Therefore, both processes must have aided transformation of aromatic precursors into aliphatic products that are less toxic, creating better living environmental conditions. Furthermore, Protobacteria, Actinobacteria, and Firmicutes are known for secreting lacasses [61] that serves as a catalyst for the rapid oxidation of phenolic and aromatic substrates. Besides, lacasses also enhance reduction of molecular oxygen to water [62, 63]. Equally, lacasses decarboxylate phenolic and methoxy-phenolic acids into nutritious compounds for bacteria [64].

Results from NMDS and PCA showed clustering of bacteria in hydrocarbon contaminated soil with plant species (T2) and one biostimulated by manure (T4), while in nonhydrocarbon contaminated soil with plant species only (T1) and one with plant species and manure (T3), the communities of bacteria were scattered from each other. The clustering could be associated with catabolic potential of dominant bacteria phyla established by this study. Although Sutton [53] reasoned that regardless of soil matrix type, clean samples (nonhydrocarbon contaminated) have higher diversity than contaminated soil, results of this study showed more diversity and clustering in hydrocarbon contaminated treatments compared to those without. The clustering must have occurred due to the ability of the treatments with TPH to selectively stimulate bacterial propagation especially through addition of carbon (mixture of aliphatic and aromatic hydrocarbons) that enriches taxa by serving as growth substrates [65]. The metabolic capacities of taxa therefore enabled biotransformation of various organic compounds by breaking down their bigger molecules into smaller units either by oxidation to release energy or complete utilization in other anabolic reactions. The versatility of taxa to use both saturated aliphatic and aromatic hydrocarbons played key role in enhancing survival and, consequently, removal of heterogeneous toxic contamination. This scenario has been observed in past studies. For example, Peng and colleagues [66] concluded that oil-polluted soils support a cornucopia of bacterial communities due to their richness in organic matter.

Addition of cattle manure to treatments with TPH increased clustering of taxa. Biostimulation with cattle manure boosted growth performance of the rhizosphere of phytoremediation plant species. The associated exudates were colonized by taxa leading to increased clustering as observed in treatments with plant species and hydrocarbon (T2) and plant species, hydrocarbon, and cattle manure (T4) (see Figure 4). This concurs with Praeg [56] that rhizosphere zones of plants are hotspots for microbial growth, abundance and diversity due to nutrient availability.

## 5. Conclusion and Recommendation

Plant species growing in TPH contaminated soil are inhabited by various strains of rhizobacteria because their roots provide excellent living conditions. In this study, rhizobacteria genera *Bacillus, Fusibacter, Luteimonas, Mycobacterium, Pseudomonas Rhodococcus,* and *Sphingomonas* were abundant in rhizosphere of phytoremediation species. In the same vein, the study also established that addition of cattle manure enhanced multiplication of these genera. Therefore, it is concluded that, in order to achieve better bioremediation, TPH contaminated soils should be bio-stimulated with cattle manure to increase rhizobacteria richness. With the exception of *Mycobacterium* (a genus that includes dangerous pathogens), this study recommends use of the genera listed above as an inoculum during ecorestoration of PHC contaminated soils in Sudd region, South Sudan. *Mycobacterium* is a carrier of tuberculosis (TB) which is a common cause of death with a prevalence rate of 257 per 100,000 people in South Sudan.

## Figures and Tables

**Table 1:** PERMANOVA analysis of interaction effects of plant species, organic manure, and hydrocarbons on bacterial community based on Bray–Curtis dissimilarity.

Factor	Df	SS	MS	F. Model	*R* ^2^	Pr (>F)
Plant species	4	0.5899	0.5987	3.9978	0.02941	0.081
Plant species: manure	1	0.9233	0.32611	3.0066	0.05130	0.009^*∗*^
Plant species: hydrocarbon	1	0.6157	0.18444	1.5462	0.09255	0.001^*∗*^
Interaction	4	0.9845	0.3217	2.2381	0.13421	0.002^*∗*^
Residuals	95	13.1882	0.13977	—	0.80211	—
Total	105	15.3171	—	—	1	—

Df = degrees of freedom; SS = sum of squares; MS = mean squares; F. Model = F-test value for model; *R*^2^ = R-squared; Pr (>F) = *p* value.

**Table 2:** Rhizobacteria community abundance and diversity in phytoremediation plant species biostimulated with cattle manure in hydrocarbon contaminated soils.

Treatments			Parameters					
Plant species	Compost (t/ha)	TPH (mg kg-1 soil)	ASVs	Number of phyla	Number of genera	Abundance chao 1	Shannon index	Simpson's index
*Gossypium barbadense*	**0**	**0**	2625	40	44	5600	4.93	0.87
		**75**	1215	24	27	2781	4. 11	0.97
	**2**	**0**	6935	101	81	14226	5.97	0.76
		**75**	4595	68.	54	9540	5.09	0.84

*Tithonia diversifolia*	**0**	**0**	3144	34	57	6638	5.14	0.82
		**75**	1936	21	34	4223	4.30	0.94
	**2**	**0**	7770	85	99	15891	6.25	0.73
		**75**	4723	60	63	9795	5.24	0.81

*Oryza longistaminata*	**0**	**0**	2764	49	66	5878	5.09	0.83
		**75**	1623	30	43	3597	4.16	0.96
	**2**	**0**	6289	99	80	12944	6.14	0.74
		**75**	4408	82	58	9168	5.16	0.82

*Sorghum arundinaceum*	**0**	**0**	2198	37	37	4745	4.86	0.88
		**75**	871	23	23	2097	3.98	0.98
	**2**	**0**	5371	69	65	11095	5.87	0.78
		**75**	3955	56	43	8260	4.84	0.89

*Hyparrhenia. rufa*	**0**	**0**	3583	79	86	7518	5.37	0.8
		**75**	1979	40	47	4304	4.54	0.92
	**2**	**0**	9675	172	164	19701	6.55	2.89
		**75**	4980	140	136	10310	5.64	0.75

**LSD (0.05) (plant** *∗ * **TPH** *∗ * **compost)**			181.1^*∗∗∗*^	**5.2** ^*∗∗*^	**2.1** ^*∗∗∗*^	**365** ^*∗∗∗*^	0.06^*∗*^	ns

^*∗∗∗*^0.000, ^*∗∗*^0.001, and ^*∗*^0.05.

## Data Availability

Data for the outputs reported in this paper are part of an ongoing Ph.D. study and can only be availed in consultation with the corresponding author reachable at janenajeb@yahoo.com.
